# Steady-state cross-correlations for live two-colour super-resolution localization data sets

**DOI:** 10.1038/ncomms8347

**Published:** 2015-06-12

**Authors:** Matthew B. Stone, Sarah L. Veatch

**Affiliations:** 1 Department of Biophysics, University of Michigan, 930 N University, Ann Arbor, Michigan 48109, USA

## Abstract

Cross-correlation of super-resolution images gathered from point localizations allows for robust quantification of protein co-distributions in chemically fixed cells. Here this is extended to dynamic systems through an analysis that quantifies the steady-state cross-correlation between spectrally distinguishable probes. This methodology is used to quantify the co-distribution of several mobile membrane proteins in both vesicles and live cells, including Lyn kinase and the B-cell receptor during antigen stimulation.

## Abstract (web summary)

Quantitative live cell super-resolution microscopy is currently limited by the time it takes to acquire a well sampled image. Here Stone and Veatch develop a cross-correlation analysis that does not rely on image reconstruction and apply this method to quantify the co-distribution of Lyn kinase and the B-cell receptor during antigen stimulation.

## Graphical abstract

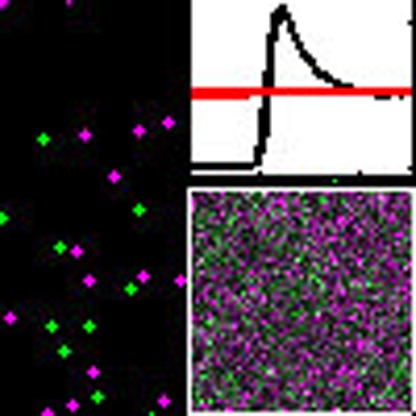

Super-resolution localization microscopy techniques such as (direct) stochastic reconstruction microscopy ((d)STORM)^1^,^2^ and (fluorescence) photoactivation localization microscopy ((f)PALM)^3^,^4^ can be used to quantitatively investigate the nanoscale co-clustering of labelled biomolecules in multi-colour fluorescence experiments. In images reconstructed from immobilized probes, the spatial co-distributions of spectrally distinct labelled proteins can be quantified using bivariate Ripley’s functions^5^ or by cross-correlation^6^,^7^,^8^. In live cells, however, single molecules can travel over large distances during the time it takes to reconstruct a single well-sampled image, complicating the interpretation of co-localization results. It is possible to quantify the co-distributions of some proteins in live cells when their dynamics are slow compared with the time it takes to reconstruct a super-resolved image^9^,^10^. However, many proteins undergo fast diffusion making this approximation invalid within the constraints of most image acquisition systems, even considering recent advances^11^ in fast image acquisition and multi-emitter fitting.

A robust set of analytical tools has been developed to quantify the time-dependent co-localization of components in diffraction-limited images based on image cross-correlation^12^,^13^, although these techniques do not take advantage of the resolution improvement afforded by localization-based super-resolution microscopy. Past work has extended cross-correlation analysis to localized single molecules through a technique named particle image cross-correlation spectroscopy^14^. While powerful, this method was designed to detect co-localization between components over short distances and reports only a single-correlation coefficient whose magnitude can vary if the density of observed components changes with time. Here we present an alternate approach that is quantitative, model-independent and robust to the variations in signal density inherent in super-resolution localization measurements. The steady-state cross-correlation analysis presented here is equivalent to approaches commonly used in statistical mechanics and condensed matter physics, which provide an estimate of the magnitude of interactions between components in units of energy, and the statistical significance of the correlation function can be estimated directly from acquired data.

Here we demonstrate the robustness of the steady-state cross-correlation approach to quantifying point localized data sets in mobile systems ranging from simulated data, an isolated plasma membrane vesicle, and live B-cell lymphocytes. Using instantaneous cross-correlation on live two-colour super-resolution data, we observe that Lyn kinase is recruited to B-cell receptors (BCRs) soon after clustering with a multivalent soluble antigen, and this recruitment is reduced but not ablated in the presence of the Src kinase inhibitor PP2. The degree of co-localization between Lyn and clustered BCR in the absence of the inhibitor corresponds to an effective interaction potential of ∼1 *k*_B_*T* over the extent of the BCR cluster (∼100 nm), where *k*_B_*T* is the thermal energy, and co-localization decreases with increased stimulation time when short (20 s) steady-state time intervals are used. By combining the steady-state cross-correlation methodology with a mobility analysis of Lyn, we find that co-localized Lyn proteins diffuse more slowly, consistent with direct binding between Lyn and immobilized components within BCR clusters. We quantify the off-rate of this interaction as well of the fraction of Lyn proteins in the immobilized state as a function of stimulation time. Overall, we conclude that this cross-correlation approach is a powerful tool to probe interactions between labelled proteins in live cell super-resolution data sets.

## Results

### Cross-correlations quantify mobile and immobile systems

The cross-correlation function, *C*(*r*, *τ*), measures the probability of finding a pair of differently coloured fluorophores as a function of their separation distance *r* at a time lag *τ*. In super-resolved data sets of immobile systems, one can quantify co-localization using *C*(*r*, <*τ*>), where <*τ*> indicates an average over all *τ*, meaning that all localizations contribute independently of when they were observed^6^,^7^,^8^. *C*(*r*,<*τ*>) is evaluated equivalently by tabulating distances between all localized fluorophores of different colours irrespective of time or by reconstructing images of each fluorophore type followed by image cross-correlation using Fourier transform methods (Supplementary Fig. 1 and Methods). Generally, the variance in *C*(*r*) determines statistical significance, depends on the number of localized molecules in each colour channel as well as the magnitude of correlations, and can be determined directly from acquired data as described in Methods and validated in Supplementary Figs 2,3. Finite localization precision as well as any errors in registering the two-image channels act to systematically broaden short-range correlations over longer distances while maintaining the integrated area under *C*(*r*)−1^15^. *C*(*r*) can be converted to the density of molecules of one type as a function of separation distance from the average labelled molecule of the other type, *ρ*(*r*), by simply multiplying *C*(*r*) by the average density of that molecule <*ρ*(*r*)>. Several methods have been described to estimate <*ρ*(*r*)> from super-resolution images^6^,^16^,^17^,^18^,^19^ or average densities can be obtained using non-imaging methods. Integrating *ρ*(*r*) allows for quantification of the average number of interacting proteins^8^,^15^. Supplementary Fig. 4 demonstrates how to convert between *C*(*r*) and *ρ*(*r*) when <*ρ*(*r*)> is known for several examples described below.

**Figure 1:**
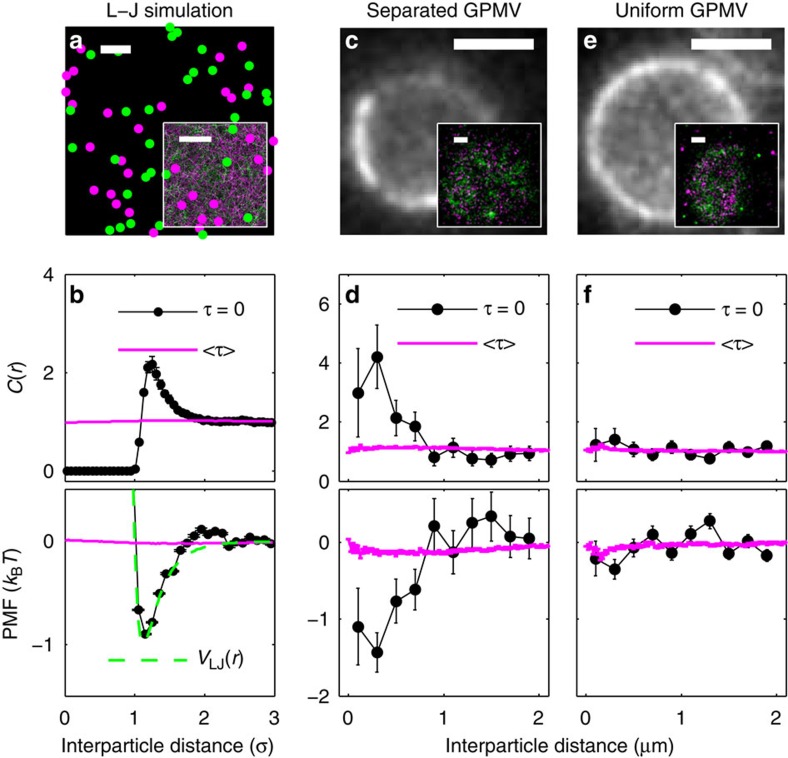
Steady-state cross-correlations quantify mobile systems. (**a**) A snap-shot of a MD simulation of the Lenard-Jones (L–J) potential. The inset shows a time-averaged reconstructed image of a subset of the simulation. Scale bar, 5σ. (**b**) The simultaneous (*C*(*r*, *τ*=0)) cross-correlation function detects more structure than the time-averaged (*C*(*r*, <*τ*>)) cross-correlation function in this mobile simulation, and the potential of mean force (PMF) tabulated from *C*(*r*, *τ*=0), but not *C*(*r*, <*τ*>), is in good agreement with the L-J potential present in this system. (**c**, **e**) Diffraction limited images of Alexa 532 CTxB bound to the surface of a GPMV with either co-existing liquid phases (**c**) or in a single liquid phase (**e**) acquired with a 0.2 s integration time. Two distinct pools of CTxB are simultaneously imaged in a STORM experiment; one conjugated to Atto 655 and the second to Alexa 532. Insets show reconstructed images of single-molecule localizations acquired from the bottom surface of vesicles over 3 min, magenta for Atto 655 and green for Alexa 532. Scale bar, 5 μm in large image and 1 μm in inset. (**d**, **f**) *C*(*r*, *τ*=0) and *C*(*r*, <*τ*>) tabulated between Atto 655 and Alexa 532 localizations for the indicated vesicles. For the phase-separated vesicle, *C*(*r*, *τ*=0) detects structure that is not apparent in the time-averaged *C*(*r*, <*τ*>) likely because domains are mobile. This corresponds to a long-range effective potential well of |PMF|>*k*_B_*T*. For the uniform vesicle (f), *C*(*r*, *τ*=0) and *C*(*r*, <*τ*>) both indicate a uniform distribution and a PMF that is indistinguishable from 0 at all radii. Error bars indicate the predicted s.d. of *C*(*r*) as calculated in equation (2).

In mobile systems, cross-correlations of reconstructed images, *C*(*r*, <*τ*>), may not reflect a meaningful co-distribution, as localizations that occur at different times are compared. This is demonstrated using a molecular dynamics (MD) simulation with particles subject to the Lennard-Jones (L-J) potential or through experimental measurements of cholera toxin B subunit (CTxB) in isolated giant plasma membrane vesicles (GPMVs) (Fig. 1). The L-J potential is strongly repulsive for short particle separations (*r*<*σ*) and weakly attractive for larger separations (*σ*<*r*<2*σ*). *C*(*r*, <*τ*>) tabulated from reconstructed images acquired over time yields a nearly uniform distribution, with *C*(*r*, <*τ*>) close to 1 for all radii (magenta line in Fig. 1b). The steady-state cross-correlation produced by tabulating *C*(*r*, *τ*=0) from pairs of particles detected simultaneously (*τ*=0) reflects the actual co-distribution of particles (black line in Fig. 1b), with exclusion at short radii (*C*(*r*<*σ*)<1) and enrichment at intermediate radii (*C*(*σ*<*r*<2*σ*)>1). A similar observation is made when tabulating cross-correlation functions between single-molecule localizations of two spectrally distinct pools of CTxB bound to GPMVs. CTxB partitions strongly into liquid-ordered domains^20^ and is highly structured in a diffraction-limited image of a phase-separated GPMV acquired with a short (0.2 s) integration time (Fig. 1c). Reconstructed images of single-molecule localizations from the bottom surface of the same vesicle appear uniform since domains are mobile over the time frame of the single-molecule measurement (3 min). *C*(*r*, <*τ*>) tabulated over all Atto 655 CTxB and Alexa 532 CTxB localizations also appears uniform; however, significant cross-correlation is observed when *C*(*r*, *τ*=0) is calculated using only pairs of probes imaged simultaneously (*τ*=0) (Fig. 1d top panel). Both *C*(*r*, *τ*=0) and *C*(*r*, <*t*>) reveal a uniform distribution of CTxB for a second GPMV that is in a single liquid phase, since both populations of CTxB explore the entire vesicle surface over both short (0.2 s) and long (3 min) time scales (Fig. 1e,f).

This quantification method also yields a model-independent measure of the effective interactions between labelled components, frequently referred to as the potential of mean force (PMF) under conditions where it can be reasonably approximated that probe organization is a result of an equilibrium process. The PMF is related to *C*(*r*) through PMF(*r*)=−*k*_B_*T*ln(*C*(*r*)), where *k*_B_*T* is the thermal energy. For the case of the MD simulation of Fig. 1, the PMF calculated using *C*(*r*, *τ*=0) reproduces the original L-J potential up to small corrections that arise from many-particle interactions (Fig. 1b, lower panel). In phase-separated vesicles, the measured potential well is greater than *k*_B_*T* out to separation distances of nearly 1 μm, consistent with the presence of phase-separated domains at thermodynamic equilibrium (Fig. 1d, lower panel). No significant PMF is observed between differently coloured CTxB in the single-phase vesicle shown in Fig. 1e,f. We note that the detailed shape of either *C*(*r*) or PMF(*r*) can be used to distinguish models describing interactions between components^6^.

### Quantifying effective protein interactions in live B cells

**Figure 2:**
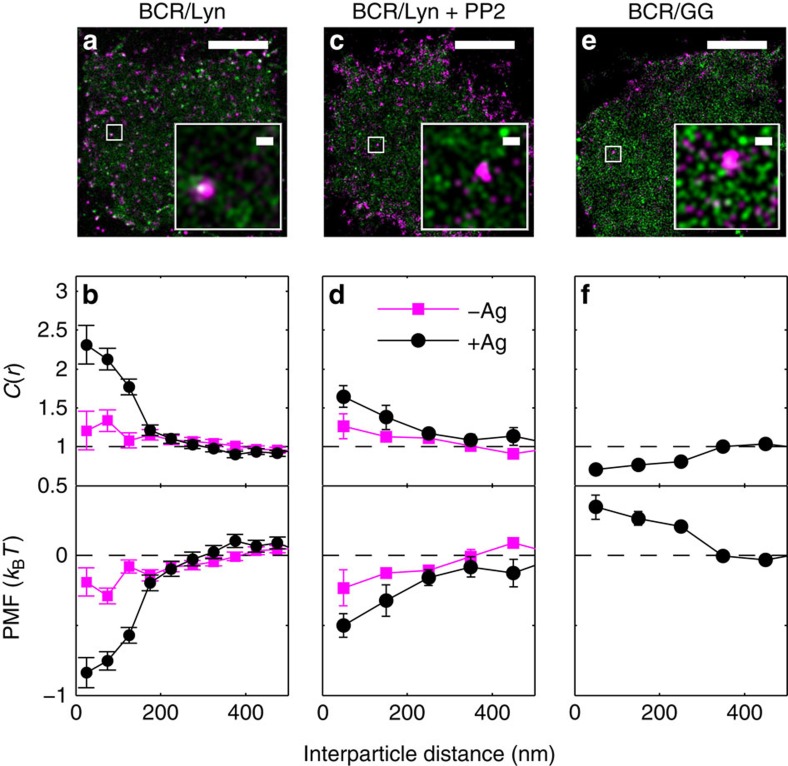
Steady-state cross-correlations in live CH27 B cells. (**a**) Lyn (green) and BCR (magenta) localizations reconstructed into an image from raw data acquired for 4 min at room temperature after the addition of 5 μg ml^−1^ streptavidin (+Ag), which clusters and stimulates BCR labelled with biotin and Atto 655. (**b**) *C*(*r*, *τ*=0) and PMF(*r*) tabulated from localizations acquired 4 min before (−Ag) or 4 min following (+Ag) antigen addition in the same cell. Error bars indicate *σC* calculated from equation (2). (**c**) Lyn and BCR imaged as in **a** but in the presence of 40 μM PP2, which reduces the magnitude of co-clustering between Lyn and BCR after antigen addition. (**d**) *C*(*r*, *τ*=0) tabulated for five distinct cells as in (**b**) then averaged to obtain statistical significance. Error bars are the s.e.m. of *C*(*r*, *τ*=0) between the five cells and *C*(*r*, *τ*=0) for all 5 cells are shown in Supplementary Fig. 5. (**e**) Reconstructed GG (green) and BCR (magenta) localizations acquired for 4 min after antigen addition. (**f**) *C*(*r*) from the cell shown in (**e**) tabulated by averaging over 0<*τ*<3 s (100 image frames) to obtain statistical significance. We do not observe *τ* dependence of *C*(*r*, *τ*), as demonstrated in Supplementary Fig. 6, likely because GG does not directly interact with BCR and most BCR clusters are not mobile over this time frame. Error bars indicate the s.e. of the weighted average as described in Supplementary Fig. 6. (**a**,**c**,**e**) All scale bars are 5 μm in large images and 200 nm in insets.

We have also applied this analysis to quantify the co-distribution of Lyn kinase and a geranylgeranylated peptide (GG) with the BCR in the CH27 cell line, which endogenously expresses an IgM isotype of the BCR (Fig. 2a). The BCR is a crucial component of the human adaptive immune system, which is able to bind a wide array of pathogen and self-epitopes. Lyn and other *src*-family kinases phosphorylate conserved tyrosine residues found in intracellular tyrosine activation motifs associated with the receptor^21^,^22^,^23^,^24^, and Lyn can bind to these phosphotyrosine residues via SH2 domains^21^,^24^. The association of Lyn with the BCR has been detected using FRET microscopy in live cells^25^. However, the initiation of BCR phosphorylation by Lyn is not fully understood. It has been suggested^26^ that Lyn is constitutively associated with BCR, or that Lyn is recruited to BCR only after antigen binding due to either a conformational change or a BCR cluster-induced stabilization of ordered lipid domains.

Lyn and BCR were, respectively, labelled with photoactivatable mEos3.2 (ref. 27) and a f(Ab)_1_ fragment conjugated to both biotin and Atto 655 (ref. 28) as described in Methods. An image reconstructed from all localized single-molecule positions acquired after the addition of soluble streptavidin indicates that BCR is strongly self-clustered under this condition (Fig. 2a), with streptavidin acting as an antigen against biotin-labelled BCR. Evaluating *C*(*r*, *τ*=0) from a single-cell data set (Fig. 2b, top panel) reveals that Lyn and BCR are weakly cross-correlated at short radii in the absence of antigen (-Ag) and correlations increase significantly in magnitude after antigen addition (+Ag), out to radii corresponding to the largest BCR clusters (200 nm). The PMF obtained from *C*(*r*, *τ*=0) (Fig. 2b, lower panel) indicates a weak attraction (|PMF|<<*k*_B_*T*) between BCR and Lyn before antigen addition, suggesting that the vast majority of Lyn is not bound to BCR in resting cells. A more significant attractive potential (|PMF|≈*k*_B_*T*) is found between these proteins after antigen addition, indicating robust Lyn recruitment to BCR clusters following antigen stimulation in agreement with previous reports using FRET^25^. We note that since our fluorophores do not distinguish between internal states of either protein, such as their phosphorylation state, the PMF should not be interpreted as the interaction strength between specific states of these proteins. Instead, it represents time and population-weighted average over all states that are present in the system.

Lyn-BCR co-clustering and the PMF between these components are reduced in cells imaged in the presence of the small-molecule *src*-family kinase inhibitor PP2 (Fig. 2c,d), also in agreement with previous FRET results^25^. PP2 inhibits phosphorylation of the BCR by *src*-family kinases, which includes Lyn, attenuating downstream signalling cascades and reducing the number and strength of potential binding sites between Lyn and BCR. Cross-correlations observed before antigen stimulation are comparable between control and PP2-treated cells, suggesting that this weak co-localization is not dependent on *src*-family kinase phosphorylation. To obtain statistically significant correlations under this experimental condition, the curves presented in Fig. 2d are generated by averaging results from five independent cells as shown in Supplementary Fig. 5.

We additionally imaged a GG conjugated to mEos3.2 simultaneously with Atto 655-labelled BCR to provide an example of exclusion in this system. GG is weakly excluded from BCR clusters formed after antigen binding, possibly due to steric repulsion from a crowded protein environment, electrostatic repulsion due to the polybasic stretch on this peptide and/or a lipid-mediated repulsion due to the disorder-preferring geranylgeranylation modification^29^, as BCR clusters are hypothesized to stabilize a more ordered local lipid environment^26^. To obtain statistical significance for this weak repulsion using data collected from a single cell, we evaluated *C*(*r*) by averaging over 0<*τ*<3s, corresponding to 100 image frames. This is appropriate in the case of antigen-clustered BCR as the partitioning of GG around BCR does not vary with τ, likely because BCR clusters do not diffuse a significant distance within this time frame. It should be noted that this averaging approach will suppress any structure in the correlation function when *C*(*r*, *τ*) varies quickly with *τ*. Additional justification for using *C*(*r*, *τ*>0) to better determine *C*(*r*, *τ*=0) in this and alternate circumstances is described in Methods and Supplementary Fig. 6.

There are quantitative differences between *C*(*r*, *τ*=0) compared with time-averaged *C*(*r*, <*τ*>) in chemically fixed samples, which can be attributed to the presence of fluorophore bleed-through (Supplementary Figs 7–9). Bleed-through occurs simultaneously in both channels; therefore, it disproportionally affects *C*(*r*, *τ*=0) compared with *C*(*r*, <*τ*>). A similar increase in simultaneous versus time-averaged *C*(*r*) is observed in simulations including a realistic level of bleed-through of mEos3.2 from the near-red to far-red emission channel, where bleed-through does not increase in the number of far-red localized positions but does bias the position of far-red localized centres (Supplementary Fig. 8). Several strategies for reducing the adverse effects of bleed-through are discussed in Methods and Supplementary Fig. 9.

### Quantifying the dynamics of the BCR-Lyn interaction

**Figure 3:**
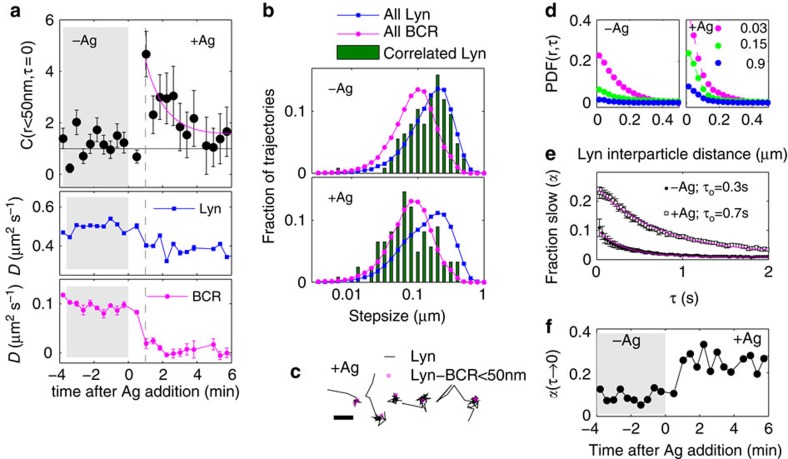
Correlation functions quantify the dynamics of BCR-Lyn co-localization in a single cell. (**a**) *C*(*r*, *τ*=0) tabulated over 20 s steady-state time intervals both before and after stimulation with multivalent antigen for the single cell. The solid magenta line is meant to guide the eye and is not a fit to any theory. Increased BCR-Lyn co-localization is coincident with reductions in both BCR and Lyn average diffusion coefficients (*D*) extracted from single-molecule trajectories as described in Methods. Error bars in the top panel indicate *σC*, as calculated from equation (2). Error bars in lower two plots indicate the s.e. in determining average *D* as described in Methods. (**b**) Distributions of BCR and Lyn displacements between sequential image frames (*τ*=0.03 s) both before (−Ag) and after (+Ag) antigen stimulation for the single cell. Bars indicate the distribution of the subset of Lyn steps adjacent to localizations correlated with BCR satisfying *C*(*r*<50 nm, *τ*=0). (**c**) Representative Lyn trajectories imaged after antigen addition show that correlated Lyn localizations (red circles) are found as trajectories are transiently confined. Scale bar, 1 μm. (**d**) Probability distribution function (PDF) for correlated displacements occurring in a time window *τ* calculated over 4 min steady-state time intervals before (−Ag) and after (+Ag) antigen addition. Curves are fit to two Gaussian shapes (equation (4)) to extract MSD(*τ*) for each population as well as the fraction in the slower population (*α*(*τ*)) as described in Methods. (**e**) *α*(*τ*) decays with time and is well fit to a single exponential *α*(*τ*)=*α*(*τ*→0)exp(−*τ*/ *τ*_o_) (magenta lines) with *τ*_o_ longer after antigen addition. Error bars indicate one s.d. in determining alpha from fits to the PDF. (**f**) An identical analysis conducted over 20-s steady-state time intervals shows the fraction of Lyn in the slowly diffusing pool as a function of stimulation time.

The simultaneous cross-correlation approach described above can also reveal time-dependent changes in protein co-localization and dynamics by tabulating *C*(*r*, *τ*=0) over shorter steady-state time intervals (every 20 s in Fig. 3a). By monitoring only correlations for *r*<50 nm versus time, it is apparent that Lyn becomes correlated with BCR shortly after antigen is added, concurrent with a marked reduction in BCR diffusion and a smaller reduction in Lyn mobility. The short-range correlations decay over 5 min following antigen stimulation, in good agreement with previous FRET results^25^, findings in chemically fixed cells (Supplementary Fig. 9) and trends are reproducible over several live cell measurements (Supplementary Fig. 10).

Lyn kinase diffuses on the inner leaflet of CH27 cells at a faster rate than the BCR; thus, a strong and long-lived binding of Lyn to BCR should be reflected as a reduced mobility of Lyn. Distributions of single trajectory diffusion coefficients show a reduction in both BCR and Lyn mobility after stimulation (Supplementary Fig. 11). We examined Lyn step-size distributions only including steps containing localized positions identified as correlated in the *C*(*r*, *τ*=0) analysis, meaning that they are observed within 50 nm of a simultaneously localized BCR. These correlated Lyn steps produce a distribution that closely resembles that observed for all Lyn steps before antigen addition, but shifts to shorter values after antigen addition to more closely resemble the distribution of all BCR steps (Fig. 3b). The majority of correlated Lyn localizations after antigen stimulation are found within sections of Lyn trajectories that are transiently confined (Fig. 3c). These findings indicate that most Lyn do not have long-lived (>0.1 s) associations with the BCR in resting cells, but a subset of Lyn becomes associated with BCR after antigen stimulation.

The same data used to tabulate spatial correlations between proteins can also be used to quantify protein mobility. The time-resolved auto-correlation function, *G*(*r*, *τ*), is tabulated from localized positions of the same fluorophore type detected at different times and is a convenient measure of the time evolution of particle motion without the need to identify single-particle trajectories. This is a localized particle variation of STICS^13^ similar to PICS^30^ but *G*(*r*, *τ*) is normalized to yield the probability density function (PDF) for correlated steps of displacement *r* in a time interval *τ*. PDF(*r*, *τ*) at fixed *τ* for a single population of diffusers is a Gaussian with width equal to the mean squared displacement (MSD). PDF(*r*, *τ*) of Lyn were fit to models containing slow and fast diffusing populations (Fig. 3d and Methods) yielding the MSD of each population and the fraction of diffusers in the slow population (*α*) as a function of *τ*. Diffusion coefficients of Lyn determined by fitting these MSD(*τ*) are in good agreement with those determined by trajectory MSD analysis (Supplementary Fig. 12). *α* decreases with *τ*, with a decay time of 0.3 s before BCR stimulation and 0.7 s following BCR stimulation (Fig. 3e). As the slowly diffusing Lyn population is likely bound to BCR or other slowly diffusing adaptor proteins, this decay likely indicates the off-rate of Lyn binding to targets associated with the BCR signalling complex. Finally, the fraction of steps belonging to the slow population extrapolated to zero time lag, *α*(*τ*→0), is the fraction of Lyn localizations associated with the slower diffusing pool. Figure 3f shows that this switches from 9±3% before antigen stimulation to 25±6% after stimulation, and does not exhibit the decay at late stimulation times observed for *C*(*r*, *τ*<50 nm) shown in Fig. 3a. This suggests that Lyn associates with other slowly moving components that are spatially distinct from BCR at later stimulation times, consistent with Lyn’s roles in phosphorylating other components during BCR stimulation such as CD19, CD22 and FcγRIIB^24^.

## Discussion

We present an analytical method to quantify the co-distribution and dynamics of labelled molecules in super-resolution localization measurements without reconstructing images or trajectories, enabling robust measurement of co-distributions in single cells exhibiting high mobility. This adds to an existing set of analysis methods based on spatial auto- and cross-correlations^12^,^13^,^14^ with specific applicability to point localized stochastically blinking probes commonly used in super-resolution fluorescence localization data sets. Here we apply this methodology to quantify interactions between membrane-bound components, but in principle this methodology could be applied to a range of systems including those imaged in one or three dimensions.

When applied to measurements of the BCR and Lyn kinase in live cells, we find that these proteins are only weakly associated before antigen stimulation, providing evidence against a model where a large fraction of Lyn is constitutively associated with BCR in the resting cell^26^. Instead, Lyn is observed to transiently co-localize with BCR soon after the addition of multivalent antigen, with single Lyn proteins dwelling with an off-rate of 0.7 s. On average, the population of Lyn associating with BCR is greatest at short times after stimulation then decreases over several minutes. This is consistent with Lyn’s role as a mediator of early signalling events and is in excellent agreement with previous findings obtained by FRET^25^. One advantage of this approach over past FRET measurements in this system is the ability to estimate interaction energies between proteins, and we observe that the potential well attracting BCR and Lyn has depth of roughly the thermal energy over a range of 100 nm. Interestingly, Lyn is still recruited to BCR clusters in the presence of the inhibitor PP2, although to a weaker extent, suggesting that there are interactions between BCR and Lyn beyond SH2 binding to phosphorylated intracellular tyrosine activation motifs within BCR. Overall, this work emphasizes the complexity of Lyn-BCR interactions as well as the power of super-resolution localization microscopy to probe the organization and dynamics of protein interactions in intact cells.

## Methods

### Calculating correlation functions

Cross-correlation functions are tabulated by first computing the distances between pairs of distinguishable localized molecules, then constructing a histogram with these distances by separating into discrete bins covering different ranges of radii, then finally normalizing this histogram to account for the different areas associated with each bin and effects that arise due to the finite size of the region of interest (ROI) being analysed. These steps are illustrated graphically in Supplementary Fig. S1b for the example of an image reconstructed from BCR and Lyn localizations in a chemically fixed cell, and are described in detail below.

Within a ROI of an image, the total number of distinct pairwise distances between distinguishable localizations is *N*=*n*_1_ × *n*_2_ where *n*_1_ is the number of localizations of one coloured probe and *n*_2_ is the number of localizations of the second coloured probe. These *N* distances are computed, and then discretized into bins centred at radii *r* with width Δ*r*, which sets the resolution of the cross-correlation. Here we call this histogram *M*(*r*). *M*(*r*) tends to increase in magnitude with increasing radius since the two dimensional area associated with each radii bin is a ring with an area of Δ*A*(*r*)=2*πr*Δ*r*. A larger associated area means that there is a greater likelihood of finding pairs separated at larger distances even if particles are randomly distributed. The total area associated with each radii bin also depends on the detailed shape of the ROI being analysed, again with larger area bins being more affected. To account for this, we assemble a normalization factor *μ*(*r*), which is the expected *M*(*r*) histogram that would be measured if all particles are uniformly distributed over the ROI: *μ*(*r*)=Δ*A*(*r*)*ρ*_0_*W*(*r*). The average density of pairwise distances over the whole area is given as *ρ*_o_=*N*/*A*_ROI_, where *A*_ROI_ is the total area of the ROI. *W*(*r*) is the radially averaged autocorrelation of the ROI that corrects for the ROI having shape that contributes to the cross-correlation, and is computed using fast Fourier transforms (FFTs) as described previously^6^. The cross-correlation function *C*(*r*) is the relative probability of finding a pair separated by a distance *r* compared with a random distribution, can then be simply expressed as:




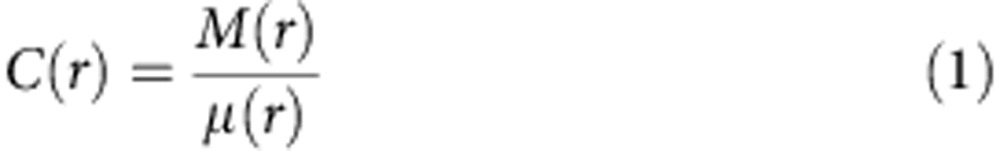




Pair cross-correlation functions describing the co-distribution of proteins can also be constructed using FFTs of the reconstructed image^6^,^8^. Supplementary Fig. 1e demonstrates that *C*(*r*) evaluated by both methods is equivalent.

The steady-state simultaneous cross-correlation, *C*(*r*, *τ*=0), is tabulated as described above, but *M*(*r*) histograms are assembled using only particle localizations that are detected simultaneously over a steady-state time interval. In this case, this histogram is normalized as above, using the total number of pairwise distances between red and green localized particles given by:




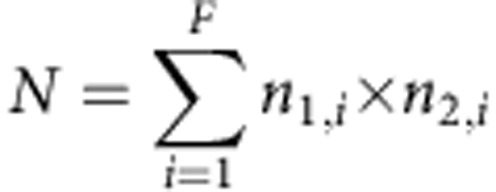




where *F* is the number of image frames included in the steady-state time window, and *n*_1,*i*_ and *n*_2,*i*_ are the number of localizations of the first and second fluorophore type in the ith image frame. *C*(*r*, *τ*) is tabulated in the same way as for *C*(*r*, *τ*=0), but by measuring pairwise distances between fluorescent localizations separated by a time interval *τ*. *G*(*r*, *τ*) measures correlations between localizations of the same fluorophore type observed with time lag *τ*. It is tabulated by the method described above for *C*(*r*, *τ*), but pairwise distances are measured between localizations of the same coloured probe. Note that *G*(*r*, *τ*=0) is a measure of the average density of probes and not a measure of self-clustering and is typically disregarded. Matlab code that tabulates *C*(*r*, *τ*) and *G*(*r*, *τ*) and associated statistical variances from point localization data is included as Supplementary Software.

### Determining statistical variance of correlation functions

Variance in *C*(*r*, *τ*) (*σ*_C_^2^) arises from statistical variance in the number of pairs associated with each spatial bin (*M*(*r*)) as well as the number of total pairs identified (*N*) over some integration time. As *M* and *N* are independent variables, the *σ*_C_^2^ can be determined through simple error propagation:




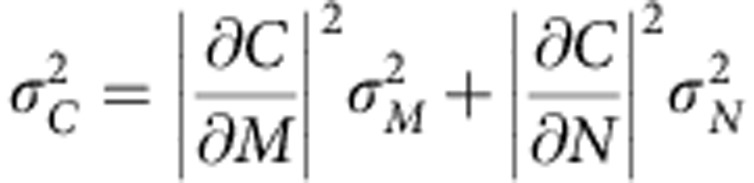




*σ*_*M*_^2^ and *σ*_*N*_^2^ are given by *M* and *N*, respectively, as is expected from Poisson counting statistics and verified in experimental data on chemically fixed cells in Supplementary Fig. 2a. For data sets with reduced sampling, it may be appropriate to approximate *σ*_*M*_^2^ as *M*+1 to provide a finite estimate of error when spatial bins contain no pairs. Using the definition of *C*(*r*) given in equation (1), this becomes




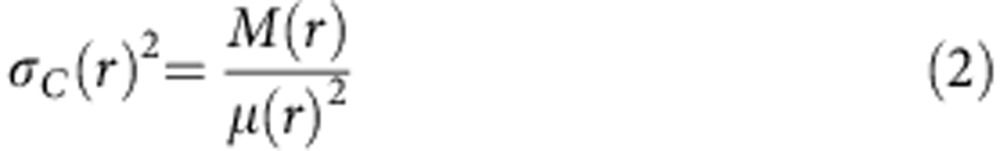




The variance in *C*(*r*) is well described by equation (2), as exemplified through data from fixed cells or resampling the MD simulation. Point localization data acquired in chemically fixed cells were re-sampled after first scrambling the time ordering of frames to remove additional contributions to *σ*_*C*_ that arise from probe photo-bleaching and/or fluorophores localizations being otherwise correlated in time. Values of *M*(*r*) and *N* were recorded and *C*(*r*,*τ*=0) was tabulated over steady-state time windows with constant number of scrambled frames. The s.d. of *M*(*r*) is well described by the square root of *M*(*r*), as shown in Supplementary Fig. 2a. In addition, the s.d. of *C*(*r*) is well described by the variance given in equation (2), which holds independent on the values of *C*(*r*), as evidenced by examining a cell chemically fixed without antigen as well as a cell chemically fixed after incubation with antigen for 1 min (Supplementary Fig. 2b). We performed a similar validation using a simulated super-resolution experiment of the Lennard-Jones MD simulation by assigning a probability that a particle is on, *P*_on_, as illustrated in Supplementary Fig. 2c. *σ*_*C*_ is estimated by resampling the simulation 15 times for each *P*_on_, changing the identity of red and green particles for each resampling. *C*(*r*, *τ*=0) is tabulated from each resampled simulation and the s.d. is used to estimate variance. We find good agreement between the measured variance and the predicted variance determined in equation (2), as illustrated in Supplementary Fig. 2d.

Using equation (2), the predicted relative error in a measurement, *σC*(*r*)/*C*(*r*), is given by




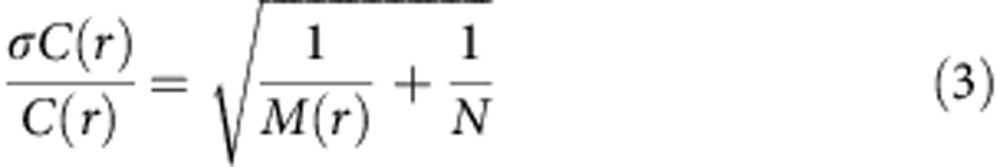




### Quantifying single-molecule mobility with correlation functions

Normalizing *G*(*r*, *τ*) such that the total two dimensional area under the curve is set to one produces a probability distribution function (PDF) describing single-molecule displacements *r* over a time lag *τ*.




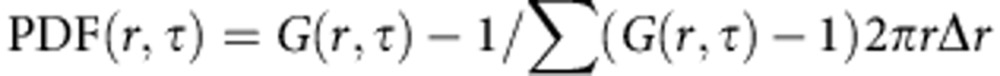




The PDF for 2 dimensional Brownian diffusion is a normalized Gaussian function with center at zero with a width equal to the mean squared displacement (MSD(τ)=<|r(t+τ)−r(t)|^2^>) which defines the diffusion coefficient, D, according to MSD(τ)=4Dτ.




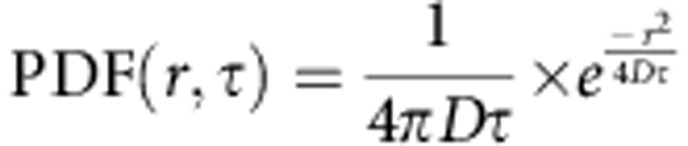




The PDF for two populations of diffusers can be written as a summation of two independent normalized Gaussians with associated diffusion coefficients *D*_1_ and *D*_2_, and a parameter *α* describing the fraction of segments associated with the diffusion coefficient *D*_1_.




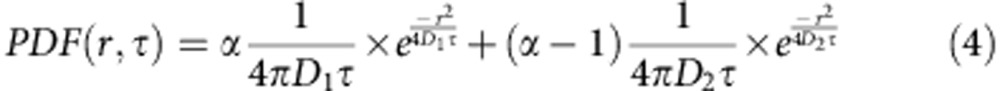




Matlab software for calculating *C*(*r*, *τ*) and *G*(*r*, *τ*) and associated statistical variances from point localization data are available online as a Supplementary Material.

### MD simulation

A MD simulation was performed utilizing the L-J potential with particle motions constrained to two dimensions. A system of 64 atoms was created with a reduced density of 0.05 atoms per unit area, giving a side length close to 36*σ*. Periodic boundary conditions were enforced, and the temperature of the simulation was adjusted by setting the initial velocities to a Gaussian distribution of magnitudes. Positions were propagated using the Verlet algorithm, and a time step of 0.005 (reduced time) was used. The starting configuration maximized space between atoms, and the simulation was allowed to equilibrate for 40,000 steps before molecular positions were tracked. A total of 200,000 time steps were taken in each simulation run, and the positions of atoms were saved every five time steps, giving 40,000 snapshots of atomic positions to use in the TRXC analysis. The atoms were randomly divided into two equal groups displayed as green and red fractions. Simulation and analysis were carried out in MATLAB (The MathWorks).

### f(Ab)_1_ and cholera toxin subunit B modification

f(Ab)_1_ fragment goat antibody to mouse IgM, μ chain specific (Jackson Immuno Research, West Grove, PA, item number 115-007-020) was simultaneously chemically modified with Atto 655 NHS ester (Sigma, St Louis, MO) and Biotin-X, SSE, 6-((Biotinoyl)Amino)Hexanoic Acid, Sulfosuccinimidyl Ester, Sodium Salt (Sulfo-NHS-LC-Biotin) (Invitrogen, Grand Island, NY). Modifications were carried out in aqueous solution buffered by 0.01 M NaH_2_PO_4_ with 0.01 M NaH_2_CO_3_, with 23 μM f(Ab)_1_ in the presence of 47 μM Atto 655 NHS ester and 140 μM Biotin–X SSE, at pH 8.2 for 30 min at room temperature. Reaction products were separated by gel filtration on Illustra NAP-5 columns (GE Healthcare, Piscataway, New Jersey) to remove unbound dye from labelled protein. Labelled f(Ab)_1_ was conjugated to additional Atto 655 in the same manner as above using 10 μM f(Ab)_1_ in the presence of 140 μM Atto 655, and unbound dye was again separated using gel filtration. Labelled f(Ab)_1_ fragment was then spun down at 20,000 × *g* for 90 min at 4 °*C* to remove any protein aggregates. We determined the average number of Atto 655 dye molecules per f(Ab)_1_ fragment to be 1.6 from absorbance measurements using a NanoDrop 2000 (Thermo Scientific, Rockford, IL). The average number of biotin per f(Ab)_1_ was determined to be 0.8 using the FluoReporter Biotin Quantification Kit (Invitrogen).

Cholera toxin subunit B (CTxB) (Sigma Alderich, St Louis, MO) modifications were carried out in aqueous solution buffered by 0.01 M Na_2_B_4_O_7_ with 0.150 M NaCl, with 19 μM CTxB in the presence of 80 μM reactive dye, either Atto 655 NHS ester or Alexa 532 NHS ester, at pH 8.2 for 30 min at room temperature. Reaction products were separated by gel filtration on Illustra NAP-5 columns (GE Healthcare, Piscataway, New Jersey) to remove unbound dye from labelled protein. Labelled CTxB was then spun down at 20,000 × *g* for 90 min at 4 °*C* to remove any protein aggregates. We determined the average number of dye molecules per CTxB to be around 3 for both Atto 655 and Alexa 532 conjugated CTxB.

### GPMV preparation

GPMVs were prepared through incubation of RBL-2H3 cells with low concentrations of dithiothreitol (DTT, 2 mM) and formaldehyde (25 mM) in the presence of calcium (2 mM) for 1 h at 37 °*C* consistent with previous work^31^. Before GPMV formation, cells were labelled with two distinct pools of CTxB for 10 min at room temperature, 0.4 μg ml^−1^ conjugated to Atto 655 and 0.1 μg ml^−1^ conjugated to Alexa 532.

### Cells, transfection and fixation

CH27 cells were a generous gift from Neetu Gupta (Cleveland Clinic, Lerner Research Institute) and were maintained in low-glucose DMEM (Life Technologies, Carlsbad, CA) containing 15% FBS (Mediatech, Manassas, VA), 10 mM HEPES, 110 mg l^−1^ sodium pyruvate, 50 μM BME and 1% Pen/Strep in 5% CO_2_ at 37 °*C*. CH27 cells were transiently expressing either Lyn protein containing a *C*-terminal fusion to the mEos3.2 photoactivatable fluorescent protein or a 20 amino-acid sequence, here called GG, coding for a polybasic stretch and *C*-terminal geranylgeranylation and N-terminal fusion to mEos3.2. A total of 500,000 CH27 cells were transfected with 0.5 μg mEos3.2-tagged plasmid DNA in Clontech N1 or C1 vector (Clontech, Mountain View, CA) using Lonza Nucleofector electroporation (Lonza, Basel, Switzerland). Plasmid DNA encoding for full-length Lyn protein and mEos3.2 have been described previously^32^, and the GG plasmid described previously^29^ was cloned to include mEos3.2. Cells were plated at 100,000  ml^−1^ and grown overnight on glass bottom wells (MatTek Corporation, Ashland, MA). Endogenous BCR in the plasma membrane was labelled with a modified f(Ab)_1_ fragment conjugated to both Atto 655 and biotin by staining with 10 μg ml^−1^ labelled f(Ab)_1_ for 10 min at room temperature in growth media followed by extensive washing with phosphate-buffered saline (Life Technologies) before imaging. Cells were stimulated by clustering f(Ab)_1_ biotin Atto 655 conjugate-labelled IgM with with 5 μg ml^−1^ soluble streptavidin. This labelling and activation scheme preserves signalling functionality under our imaging conditions, as indicated by increased tyrosine phosphorylation and calcium mobilization after the addition of antigen (Supplementary Fig. 3). For fixed cells, CH27 cells were transfected with Lyn-mEos3.2 and BCR was stained with modified f(Ab)_1_ fragment as described above before holding in control buffer (135 mM NaCl, 5 mM KCl, 1 mM MgCl_2_, 1.8 mM CaCl_2_, 5.6 mM glucose, 20 mM HEPES) at room temperature during addition of streptavidin (Life Technologies) at 5 μg ml^−1^ to stimulate cells. Cells were then washed extensively in phosphate-buffered saline before chemical fixation with 4% formaldehyde and 0.01% gluteraldehyde (Ted Pella Inc, Redding, CA) for 10 min at room temperature.

### TIRF microscopy

Imaging was performed on an Olympus IX81-XDC inverted microscope with a cellTIRF module, a 100 × UAPO TIRF objective (NA=1.49), active Z-drift correction (ZDC) (Olympus America, Center Valley, PA). Images were acquired on an iXon-897 EMCCD camera (Andor, South Windsor, CT). Excitation of Atto 655 was accomplished using either a 647-nm diode laser for live-cell measurements (OBIS 647 LX-100FP, Coherent, Santa Clara, CA) or a 641-nm diode laser for GPMV measurements (CUBE 640-75FP, Coherent). Excitation of Alexa-532 was accomplished using a 532-nm diode laser (150 mW Samba, Cobolt, Sweden), and excitation of mEos3.2 constructs was accomplished using a 561-nm diode laser (Sapphire 561 LP, Coherent). Photoactivation of mEos3.2 was accomplished with a 405-nm diode laser (CUBE 405-50FP, Coherent). Laser intensities were adjusted such that single fluorophores could be distinguished in individual images. Excitation and emission were filtered using the quadband filter cube set ET-405/488/561/647 (Chroma, Bellows Falls, VT) for both mEos3.2/Atto 655 and mEos3.2/Dyomics 654 fluorophore pairs or filtered using ET-405/488/532/640 for Alexa 532/Atto 655 fluorophore pair. Emission was split into two channels using a DV2 emission splitting system (Photometrics, Tuscon, AZ) using a T640lpxr dichroic mirror to separate emission, ET605/52m to filter near-red emission, and ET700/75m to filter far-red emission (Chroma).

Live cells were imaged in a live cell compatible imaging buffer: 30 mM Tris, 100 mM NaCl, 5 mM KCl, 1 mM MgCl_2_, 1.8 mM CaCl_2_, 50 mM glucose, 12 mM glutathione, 40 μg ml^−1^ catalase (Sigma), 500 μg ml^−1^ glucose oxidase (Sigma), pH 7.5. Where noted, cells were treated with 40 μM PP2 (Invitrogen) in live cell compatible imaging buffer for 5 min before and during antigen stimulation. Fixed cells were imaged in buffer with higher buffering capacity, glucose concentration and pH: 50 mM Tris, 550 mM glucose, 10 mM NaCl, 12 mM glutathione, 40 μg ml^−1^ catalase, 500 μg ml^−1^ glucose oxidase, pH 8.5. GPMVs were diluted 1:1 into live cell compatible imaging buffer. A small quantity of water (5%) was also added to better match the osmolarity of the GPMV and imaging buffers. GPMVs were imaged using off-TIR excitation between two #1.5 coverslips with a vacuum grease spacer and attached to a home-built Peltier-based temperature stage coupled to a PID controller (Oven Industries, Mechanicsburg, PA), consistent with previous work^31^.

### Western blots and Ca^2+^ mobilization

CH27 cells were incubated with 10 μg ml^−1^ of goat anti-mouse IgM μ-chain specific f(Ab)_1_-biotin fragment (Jackson) for 10 min to label the IgM with biotin and then subsequently washed twice by centrifugation. One million CH27 cells were each suspended in either control buffer (defined above), live cell imaging buffer (defined above) or imaging buffer without oxygen scavenging enzymes glucose oxidase and catalase. Some cells were stimulated by the addition of 5 μg ml^−1^ streptavidin. Cells were lysed at room temperature using RIPA buffer (EMD Millipore, Billerica, MA) in the presence of Halt Phosphatase Inhibitor Cocktail (Thermo Scientific) and Complete Mini Protease Inhibitor Cocktail (Roche, Basel, Switzerland). Cell lysates were centrifuged at 16,000 × *g* for 15 min at 4 °*C*. Supernatants were run on a denaturing sodium dodecyl sulfate–PAGE (SDS-PAGE) gel, 7.5% Mini-PROTEAN gel (Bio-Rad, Hercules, CA) and then transferred to an Immobilon-P PVDF transfer membrane with 0.45 μm pore size (Millipore). Blots were stained with a 1:2,000 dilution of mouse IgG2b 4G10 Platinum anti-phosphotyrosine antibody (Millipore, catalogue number 05-321) and subsequently stained with a 1:5,000 dilution of peroxidase-conjugated goat anti-mouse IgG2b specific secondary antibody (Jackson Immuno research, catalogue number 115-035-207). Blots were sensitized using SuperSignal West Pico chemiluminescent substrate and developed on Amersham Hyperfilm ECL (GE Healthcare Biosciences, Piscataway, NJ). Ca^2+^ mobilization assays were performed by incubating three million CH27 cells per ml with 2 μg ml^−1^ Fluo-4, AM (Life Technologies) in the presence of 0.25 mM sulfinpyrazone for 5 min at room temperature and then diluting to 0.2 million cells per ml in the same buffer for 30 min at 37 °*C*. Cells were washed twice by centrifugation and resuspended in either control buffer or imaging buffer. CH27 IgM was labelled with biotin as above and then cells were loaded into wells 96-well black plates at a concentration of two million per ml, and fluorescence was assayed by exciting the cells with 485 nm light and collecting 520 nm light in the Omega PolarStar (BMG Labtech, Ortenberg, Germany).

### Single-molecule analysis

Single-molecule fluorescent events were localized by fitting local maxima in background subtracted images to Gaussian functions, and images were reconstructed^6^. In brief, background subtracted raw images were bandpass filtered and local maxima above a threshold were used as starting locations for two-dimensional Gaussian fitting to unfiltered background subtracted images. The width and errors of the Gaussian fits as well as the sum of intensity in the fluorescent spot were used to cull outliers in each distribution of parameters. Stage drift was corrected for every 500 frames by finding the maximum in the 2D cross correlation produced by all localizations between successive groups of frames.

Localizations in the near-red channel were registered with the far-red channel using fiducial markers with adapted methodology^33^. In brief, 100 nm diameter Tetraspeck beads with fluorescence emission in both near- and far-red channels (Invitrogen) were adhered to glass slides, excited by both 561 nm (or 532 nm) and 647 nm lasers, and 70 fluorescent images of 20–40 beads were collected before and after the acquisition of each data set. These diffraction-limited fluorescent beads were used as control points to create a polynomial transform from the near-red channel to the far-red channel, and this polynomial transform was applied to mEos3.2 localizations in the near-red channel.

Stage-drift-corrected and emission channel-registered point localizations were used to reconstruct a multicolour super-resolution image by incrementing pixels corresponding to 25 nm for each localization falling into that pixel for each emission channel. *C*(*r*,<*τ*>) was determined from images, *I*_1_ and *I*_2_, reconstructed from point localizations collected from each emission channel over time as follows consistent with previous work^6^.




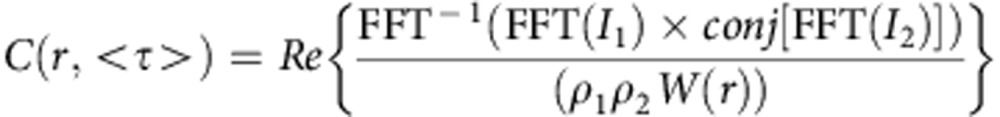




Here, *conj[]* indicates a complex conjugate, *ρ*_1_ and *ρ*_2_ are the average surface densities of images *I*_1_ and *I*_2_, respectively, and Re{} indicates the real part. *C*(*r*,*τ*) was determined using stage drift-corrected and emission channel-registered point localizations. Both *C*(*r*, *τ*) and *C*(*r*, <*τ*>) were computed from point localizations falling within a user-defined ROI, and this ROI was used to determine normalization factor *W*(*r*).

Single-molecule trajectories were determined using a tracking algorithm that searches for localizations within 500 nm in subsequent frames and terminates ambiguous trajectories^34^. The average MSD as a function of time interval (*τ*) was tabulated for all trajectories and diffusion coefficients were extracted through a linear fit to the second through fourth time point of the MSD(*τ*), and error bounds reflect s.e. determined directly from this fit to determine the average diffusion coefficient *D*. Single trajectory diffusion coefficients were obtained by tabulating MSDs from single long trajectories (greater than 10 segments), and fitting the second through fourth point of MSD versus *τ* in the same manner to obtain individual diffusion coefficients D.

### Strategies to reduce the effects of bleed-through

As demonstrated in Supplementary Figs 7, 8, and 9, fluorescent bleed-through of one fluorophore into the other image channel leads to additional spatial correlations at short distances that are also correlated in time. While the most reliable method to reduce the adverse effects of bleed-through are to choose probe pairs that minimize this artefact, several analytical approaches can be used to correct for bleed-through under specific circumstances. For example, if dynamics are slow compared with both the acquisition time for single-molecule detection and the characteristic on-time of fluophoroes, then it is possible to extrapolate *C*(*r*, *τ*) to *τ*=0 while excluding the small *τ* points that are affected by bleed-through. An application of this can be seen in Supplementary Fig. 6, where *C*(*r*, *τ* =0) is systematically higher than *C*(*r*, *τ* → 0) for the first spatial bin (*r*<50 nm for Lyn and *r*<100 nm for GG). For instances where co-localization dynamics are fast compared with acquisition time or fluorophore on-times, then it may be possible to estimate the magnitude of a bleed-through correction to *C*(*r*, *τ*=0) by first calibrating with a fixed cell sample, where the effects of bleed-through can be directly measured (Supplementary Fig. 9). Under conditions where it can be assumed that bleed-through properties are not altered by fixation, then in principle this correction could be applied to live cell data. It should be noted that chemical fixation can alter the quantum yield of some fluorophores^35^, so there is limited applicability of this method. Finally, it may be possible to correct for bleed-through directly, by first measuring the magnitude of the bleed-through signal, then subtracting the predicted bleed-through signal directly from acquired data before image processing. In our hands, we found this method to be computationally expensive, as it requires that fluorophores be first localized, then a spatial transform computed to properly localize the bleed-through signal on the second image channel. We also found this method to be only moderately effective for reducing the magnitude of *C*(*r*, *τ*=0) in cases of known bleed-through, likely due to the uncertainty in intensity and localization inherent to this treatment.

### Code availability

Matlab code for calculating correlation functions is available as Supplementary Software through Nature Communications website.

## 

## Additional information

**How to cite this article:** Matthew B. Stone and Sarah L. Veatch. Steady-state cross-correlations for live two-colour super-resolution localization data sets. *Nat. Commun.* 6:7347 doi: 10.1038/ncomms8347 (2015).

## Supplementary Material

Supplementary Information Supplementary Figures 1-13.

Supplementary SoftwareA MATLAB function to compute C(r,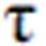) and G(r,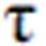) from super-resolution point localization data sets.
