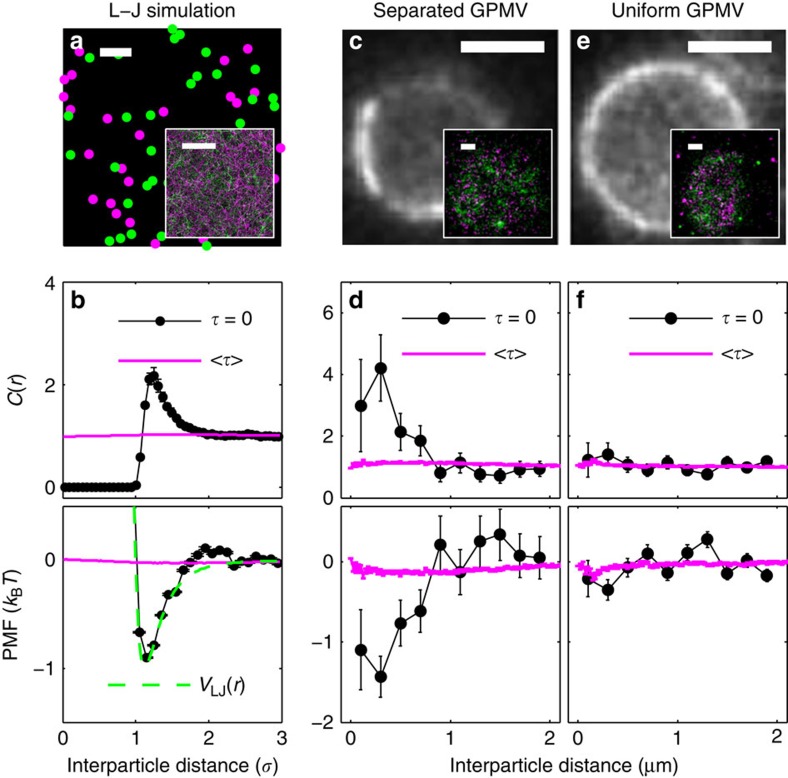# Erratum: Steady-state cross-correlations for live two-colour super-resolution localization data sets

**DOI:** 10.1038/ncomms9319

**Published:** 2015-09-08

**Authors:** Matthew B. Stone, Sarah L. Veatch

*Nature Communications* 6: Article number: 7347 10.1038/ncomms8347 (2015); Published 06122015; Updated 09082015

This Article contains an error in Fig. 1 that was introduced during the production process. The inset in panel a should appear on top of the green and magenta dots, not beneath them. The correct version of the figure appears below.

## Figures and Tables

**Figure 1:**