# Supplementary material for: Steady-state cross-correlations for live two-colour super-resolution localization data sets
Source: Nat Commun. 2015 Jun 12;6:7347. doi: 10.1038/ncomms8347 (PMC4467025; doi:10.1038/ncomms8347)
Supplement: Supplementary Information — Supplementary Figures 1-13. [file ncomms8347-s1.pdf]

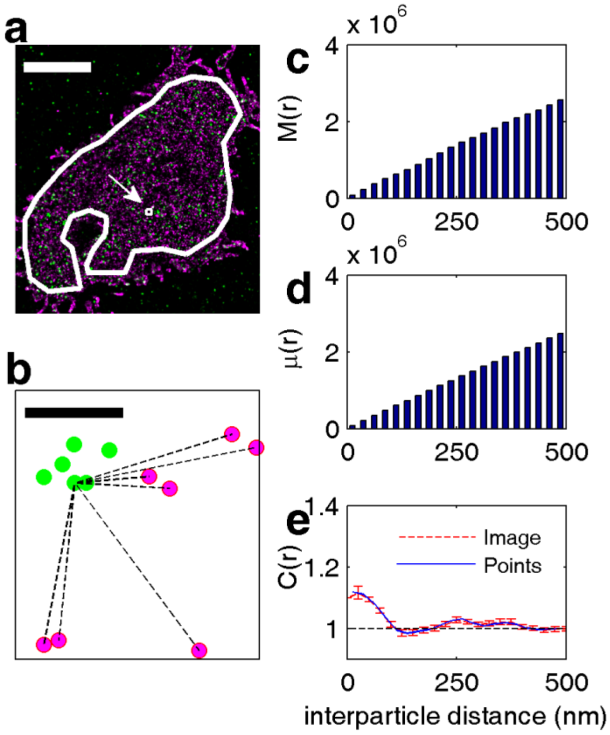

Supplementary Figure 1: Construction of point by point cross correlation and equivalence to FFT method. (a) A reconstructed image of a chemically fixed B cell showing Lyn-mEos3.2 localizations in green and BCR Dyomics 654 localizations in magenta. Point-by-point and image cross correlations were evaluated within the region of interest (ROI) outlined in white pointed to by white arrow. Scale bar is 5µm. **(b)** Point-by-point cross-correlations are tabulated by first measuring the distance between all possible pairs of red and green localizations. The sub-region denoted by small white box in (a) is used as an example and the distances between a single green particle and all magenta particles in this sub-region are shown as dotted lines for demonstration purposes. Scale bar is 100nm. **(c)** The histogram M(r) constructed by binning all observed interparticle distances with a uniform bin width of Δr=25nm. **(d)** The normalization factor µ(r) is the expectation value for the number of interparticle distances observed for each bin in M(r) given a random distribution. **(e)** The point-by-point cross-correlation function C(r) is determined by dividing the observed interparticle distance histogram M(r) by the normalization factor µ(r). Thus, C(r) indicates that the observed interparticle distances is a random distribution when C(r) is equal to 1. Tabulating the cross-correlation using fast Fourier transforms (FFTs) on reconstructed images, shown in red, agrees with the point-by-point method, shown in blue from the same acquired data.

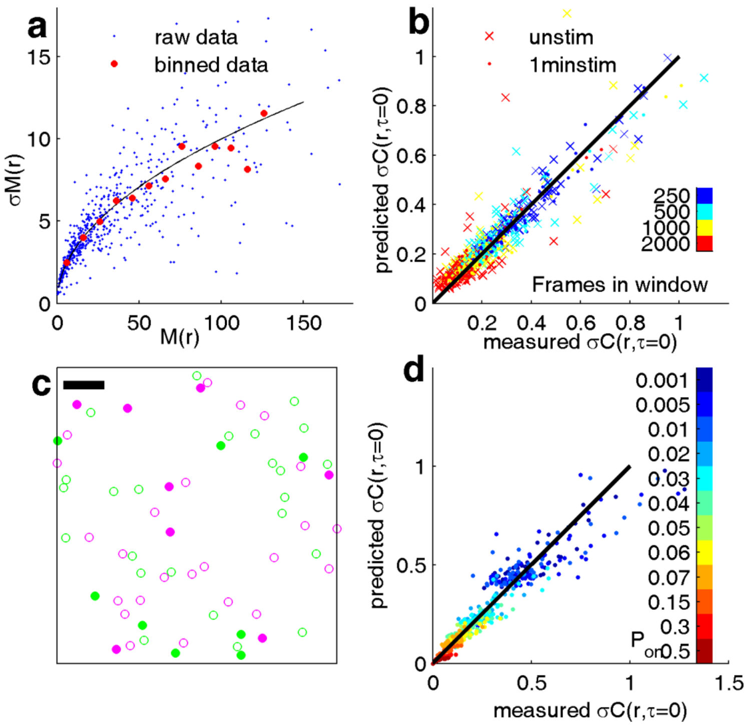

Supplementary Figure 2: Verification of statistical errors in determining C(r). **(a)** The standard deviation of M(r), σM(r), as a function of the average value of M(r), <M(r)>, was determined from point localization data from 7 distinct chemically fixed cells labeled with BCR and Lyn, where single molecules were imaged over an average of 9000 frames per cell. This dataset included both antigen stimulated and unstimulated cells. In order to probe a broad range of <M(r)> values, M(r) was tabulated with Δr=100 nm spatial bins using time windows that were shorter than the total number of frames acquired during an experiment. Time windows of equal size; 250, 500, 1000, or 2000 frames were compared on a cell-by cell basis, and σM(r) vs. <M(r)> is plotted for all time windows and radii bins in all cells as blue dots. Larger red circles show average values of M(r) over a range of <M(r)> values (<M(r)> ±5). The black line is the square root of M(r), illustrating that σM(r) varies as $<M(r)>^{0.5}$. **(b)** C(r) was determined from small time windows for the fixed cell data described in (a). The σC(r) between time windows of equal size for individual cells was measured and the error predictor σC(r) was calculated from Eqn. 2 using average values of <μ(r)> and <M(r)> between time windows as in (a). The black line is of slope 1 through the origin, showing agreement between the predicted error and the measured and error obtained from repeated measurements. **(c)** A representative frame from a down-sampled Lennard-Jones (L-J) molecular dynamics (MD) simulation in which only 25% of positions are 'on', illustrated as filled circles, and all other particles are 'off', illustrated as empty circles. Only particles that are 'on' contribute to determining C(r, τ=0). Scale bar is 5σ. **(d)** MD simulation frames were down-sampled at various rates with probability of a particle being on as P_{on}, and sampling for each P_{on} was repeated 15 times with new balls being designated as magenta or green in each repeat. Particles blinked 'on' for 3 frames at a time and blinking was randomized between repeats. C(r, τ=0) was determined from particles on in the same frame using a bin size of Δr=0.061σ, and the standard deviation of all spatial bins of C(r, τ=0) between repeats were determined for all P_{on}. The error predictor σC(r) was calculated using Eqn. 2 in Methods. Colors indicate various P_{on} and the black line is of slope 1 through the origin, showing agreement between the predicted error and the measured and error obtained from repeated measurements.

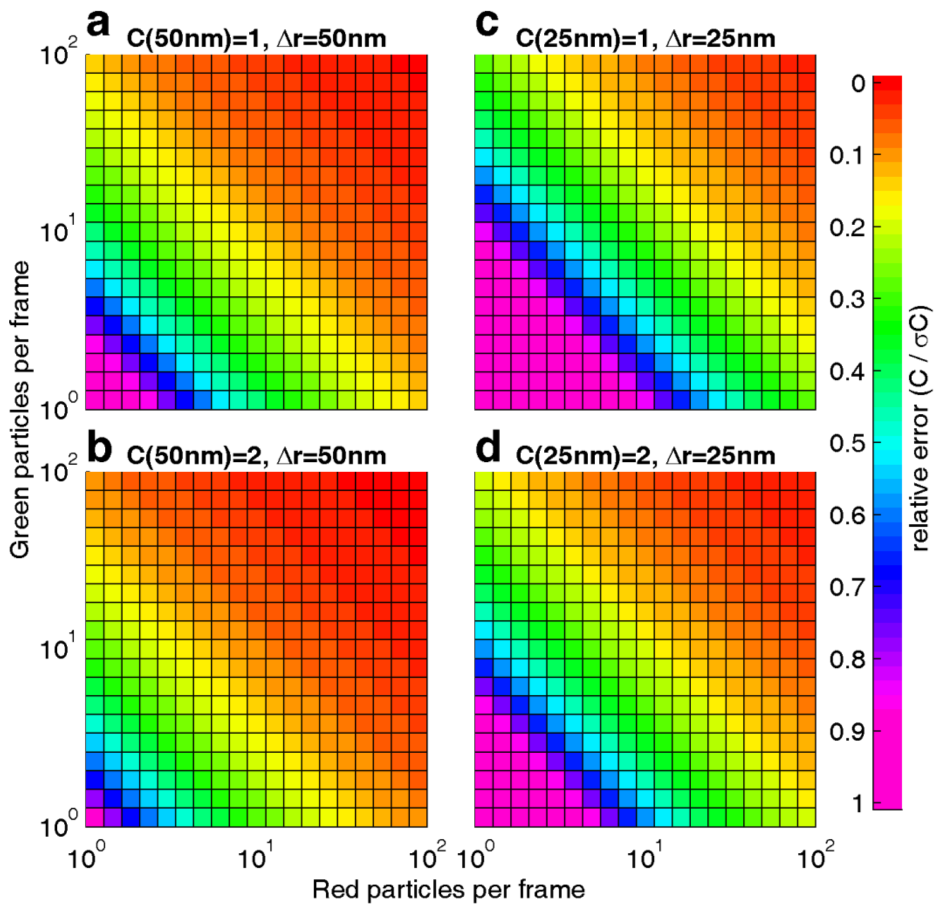

Supplementary Figure 3: Statistical errors in C(r) are a function of the number of objects detected as well as the spatial bin size, Δr, and the observed correlation function. The surface plots above show how σC(r, τ))/C(r, τ) given by Eqn. 3 varies with the number of localized particles per frame collected over 5000 frames, obtained by using M(r, τ)=C(r, τ)μ(r) and set values for C(r, τ) into the expression for σC(r, τ)/C(r, τ) given in Eqn. 3. We use a 10μm diameter circular mask and either 25nm or 50nm bin size to calculate W(r) and ΔA(r), and here we analyze only the first point of C(r). Relative error is given by the colorbar at right and relative error > 1 is not distinguished here. **(a,b)** Surface plot of predicted σC(r<50nm)/C(r<50nm) assuming either a random distribution (C(r<50nm) = 1) or a weakly co-clustered distribution (C(r<50nm) = 2). **(c,d)** Surface plot of predicted σC(r<25nm)/C(r<25nm) assuming either a random distribution (C(r<25nm) = 1) or a weakly co-clustered distribution (C(r<25nm) = 2). Note that increased spatial resolution requires greater sampling to obtain the same level of statistical significance. Also, optimal sampling is obtained when the same number of localizations are detected in each color channel, but poor sampling in one channel can be compensated for by greater sampling in the second channel.

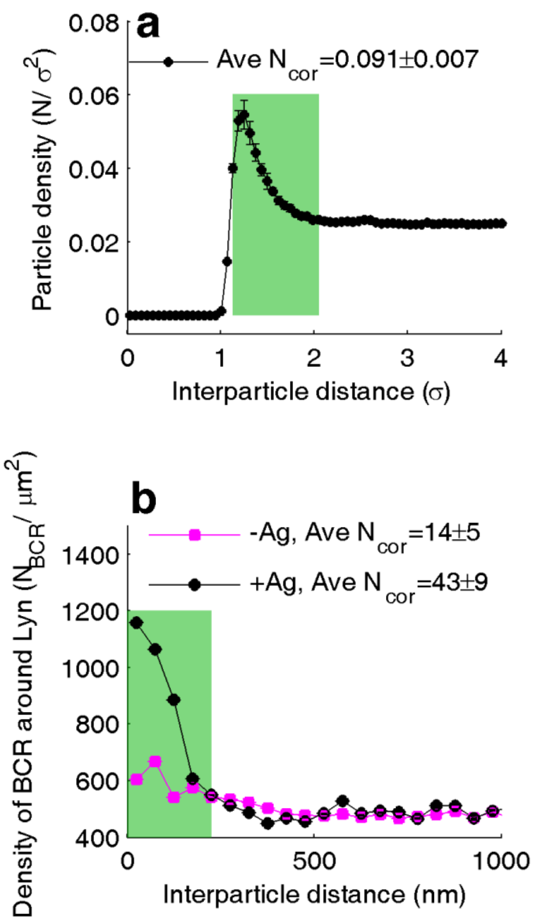

Supplementary Figure 4: Particle density as a function of interparticle separation. If the average particle density (<ρ >) is known, then C(r) can be converted to the density of that type of particle as a function of separation distance from other particle type through ρ(r)=<ρ>C(r). **(a)** For the L-J MD simulation, <ρ> for both red and green particles is 0.025 particles / σ^2. Multiplying C(r) by this density gives ρ(r) between red and green particles. The particles are correlated between 1.1σ and 2σ where the attractive component of the L-J potential dominates, and the average number of correlated particles, N_{cor}, can be determined by measuring the area under the curve: $N_{cor} = \rho \sum (C(r) - 1) 2\pi r \Delta r$ over the range of correlations. In this case, N_{cor} is less than 1 since the particle density in the simulation is low and the interaction is weak. **(b)** For the case of Lyn and BCR in a live cell, the average density of BCR can be estimated from previous work[1,2] to be 500 BCR / μm^2. Multiplying the observed C(r) from the single live cell shown in **Fig. 2 a, b** by this BCR density yields the density of BCR proteins as a function of separation distance from the average Lyn protein. These proteins are correlated with each other between 0 and 225 nm. $N_{cor}=\rho \sum (C(r) - 1) 2\pi r \Delta r$ is evaluated over this area gives the values shown in the figure legend.

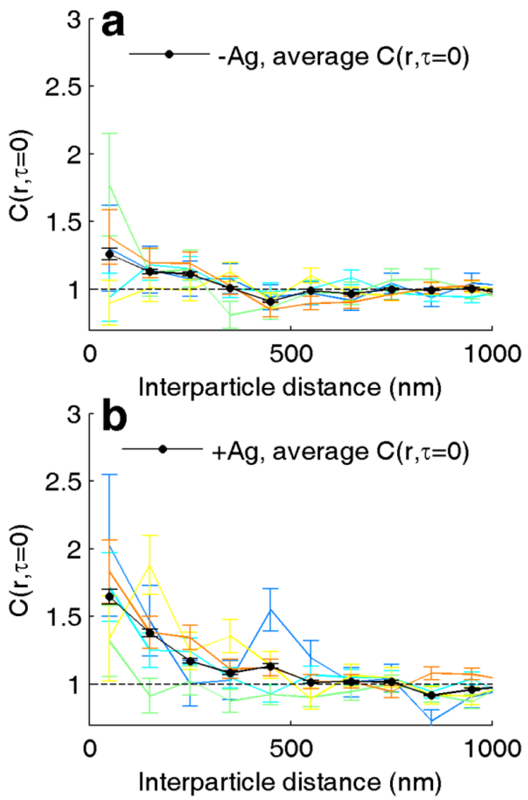

Supplementary Figure 5: Averaging C(r, τ=0) between cells treated with PP2. Cells expressing Lyn-mEos3.2 and having BCR stained with Atto 655 were pretreated with PP2 for 5 minutes and then stimulated while performing a two-color super-resolution experiment in which the positions of BCR and Lyn were simultaneously collected. Data was collected from 5 cells, with 5000 frames of data collected before antigen stimulation (-Ag) and 5000 frames of data collected after antigen stimulation with streptavidin (+Ag). **(a)** C(r, τ=0) curves for 5 cells prior to antigen stimulation. Data from individual cells are plotted as colored lines with error bars given by σC and the average of all 5 cells is plotted as a black line with error bars corresponding to the standard error of the mean between the 5 cells. **(b)** C(r, τ=0) curves for 5 cells following antigen stimulation. Data from individual cells are plotted as colored lines with error bars given by σC and the average of all 5 cells is plotted as a black line with error bars corresponding to the standard error of the mean between the 5 cells. Although individual C(r, τ=0) curves are noisy, averaging individual curves together allows for more reliability when samples can be directly compared.

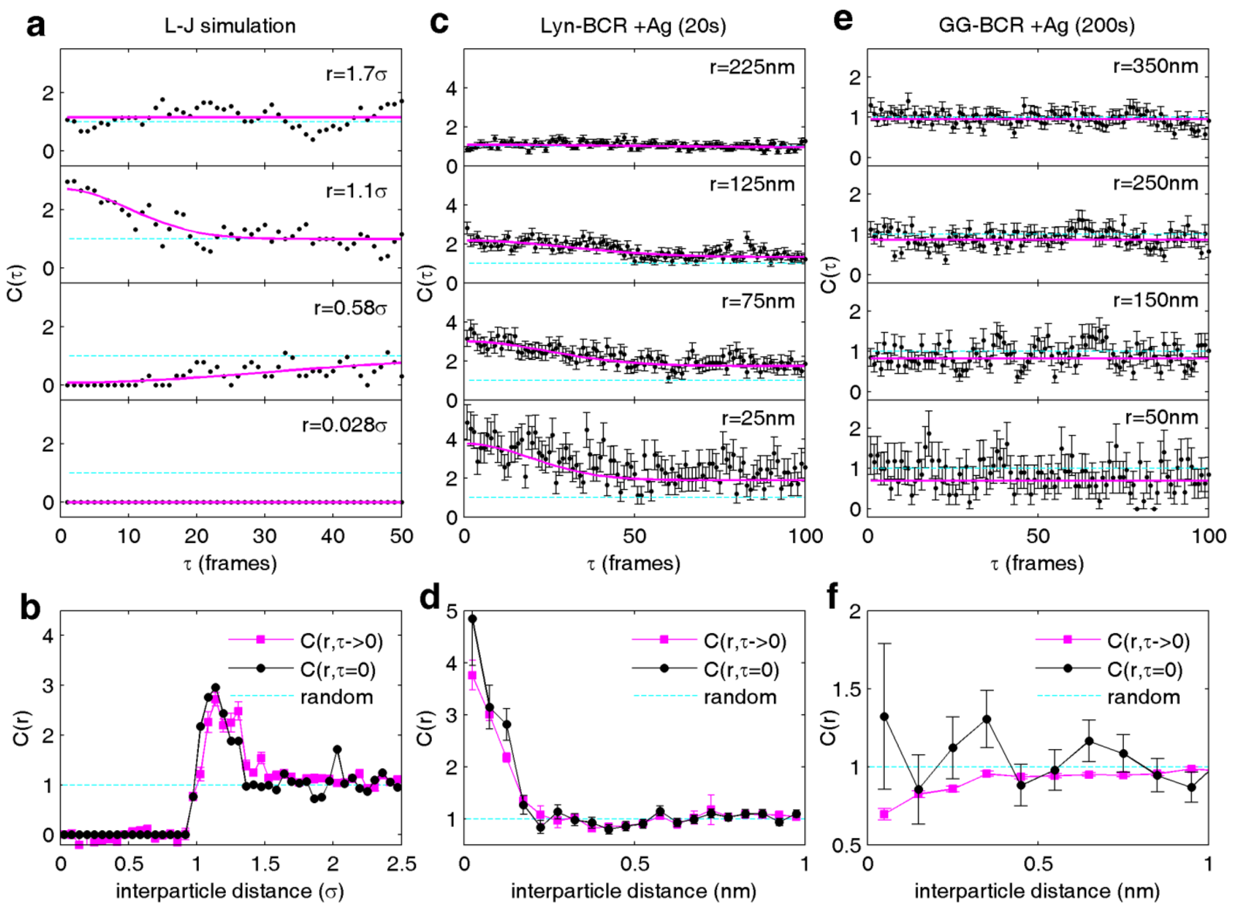

Supplementary Figure 6: Using C(r, τ) to better define C(r, τ=0). Tabulated values for C(r, τ) can be used to better specify the value at τ=0 if the dynamics of colocalization are slow compared to the frame rate of acquired data. **(a)** This approach is validated in MD simulations of the L-J potential, where C(r, τ) varies over tens of frames. Here, C(r, τ) was fit to a Gaussian shape in τ (magenta lines). **(b)** The best fit line evaluated at τ=0 (C(r, τ→0) is in good agreement with C(r, τ=0). **(c)** C(r, τ) for several r values tabulated from 500 image frames soon after addition of antigen for the same cell presented in Figs 2a and 3 with labeled BCR and Lyn. C(r, τ) varies slowly with τ and again is fit with a Gaussian shape, with weights given by $1/\sigma C^2$ (magenta lines). **(d)** The extrapolated C(r, τ→0) is in good agreement with the raw C(r, τ=0) but exhibits better signal to noise, where error bounds indicate one standard deviation in the Gaussian fit parameters. **(e)** C(r, τ) at several r values tabulated from 5000 frames after antigen addition for the cell shown in Fig 2e labeled GG and BCR. Interestingly, C(r, τ) does not decay with τ over 100 image frames (τ<3s). The magenta lines represent a weighted average over all 100 points, with weights given by $w=1/\sigma C^2$ (magenta lines). **(f)** The weighted average over τ produces a C(r, τ=0) that is much better specified than the raw C(r, τ=0), with error bounds given by $(1/\sum w)^{0.5}$. Note that in d and f, raw C(r, τ=0) are systematically higher than extrapolated C(r, τ→0) in the first spatial bin. This likely is a consequence of bleed-through between image channels, which only affect C(r, τ) at very short τ.

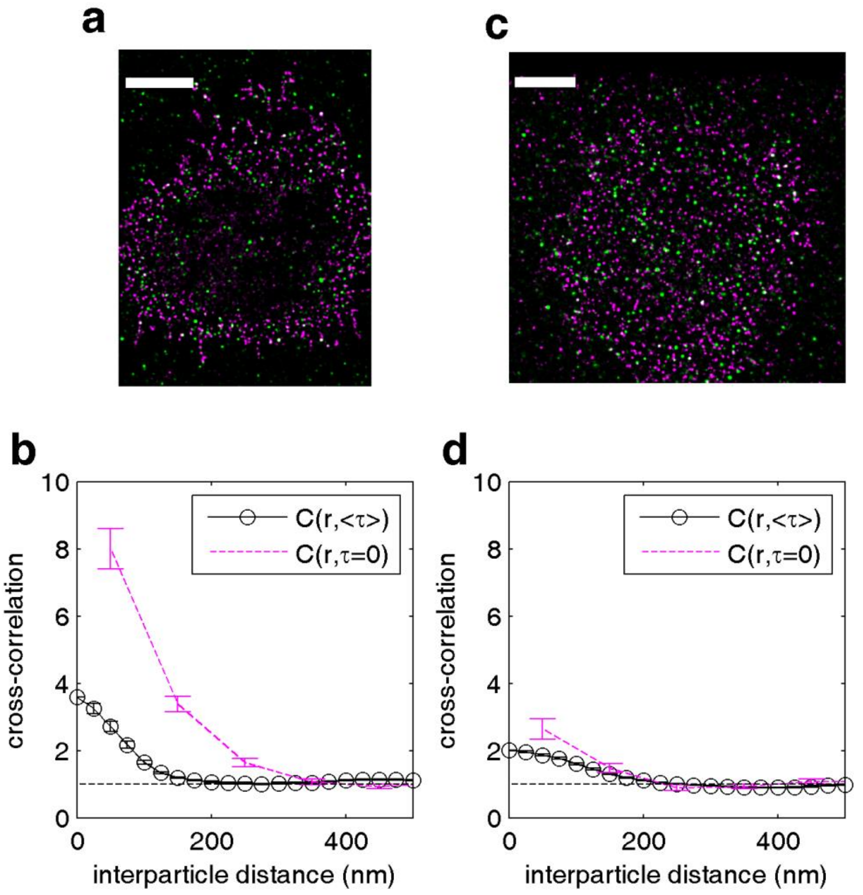

Supplementary Figure 7: Overestimation of C(r, τ=0) in chemically fixed cells depends on probe pairs used. **(a)** Reconstructed super-resolution image from simultaneous two-color imaging with Dyomics 654 labeled BCR and mEos3.2 labeled Lyn in a cell stimulated for one minute before chemical fixation. Magenta pixels represent Dyomics 654 localizations and green pixels represent mEos3.2 localizations. Scale bar is 5 µm. **(b)** Cross-correlation functions calculated from localized positions within the cell footprint. C(r, <τ>) is calculated by comparing all localizations in the entire experimental dataset, and C(r, τ=0) is calculated from only localizations that are imaged simultaneously in the same acquisition frame. Dramatic increases in C(r, τ=0) vs. C(r, <τ>) indicate that signals in the two channels are highly correlated in time, most likely due to bleed-through between spectral channels for this probe pair. **(c)** Reconstructed super-resolution image from simultaneous two color imaging with Atto 655 labeled BCR and mEos3.2 labeled Lyn in a cell stimulated for one minute before chemical fixation. Magenta pixels represent Atto 655 localizations and green pixels represent mEos3.2 localizations. Scale bar is 5 µm. **(d)** Cross-correlation functions calculated from localized positions within the cell footprint. C(r, <τ>) is calculated by comparing all localizations in the entire experimental dataset, and C(r, τ=0) is calculated from only localizations that are imaged simultaneously in the same acquisition frame. Error bars in (b) and (d) reflect σC. While C(r, τ=0) remains slightly elevated compared to C(r, <τ>) with the Atto 655 and mEos3.2 probe pair, it is reduced when compared to cells labeled with Dyomics 654 and mEos3.2, justifying the use of Atto 655 in these measurements.

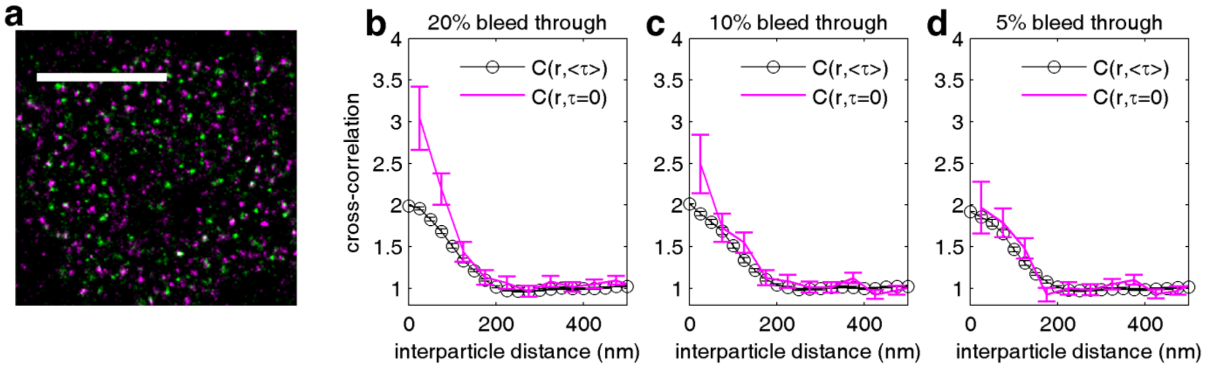

Supplementary Figure 8: Simulated datasets confirm that bleed-through increases C(r, τ=0) while leaving time-averaged cross-correlations unaffected. **(a)** An experimental co-distribution of Lyn (green) and BCR (magenta) was determined from two-color super-resolution of a chemically fixed CH27 cell where the BCR was labeled with Atto 655 and Lyn was labeled with mEos3.2. Scale bar is 5µm. **(b-d)** Steady state (C(r, τ=0)) and time-averaged (C(r, <τ>)) cross-correlation functions tabulated from simulated data with the specified bleed-through of mEos3.2 fluorescence emission intensity into the far-red acquisition channel with error bars given by σC. Simulated data was generated by randomly selecting localized positions to blink on and off with intensities and off-times sampled from experimentally determined distributions. Fluorophore fluorescence was simulated from experimental localizations using a diffraction limited point spread function, and additionally superimposing mEos3.2 bleed-through intensity at the specified level in the far-red channel. White noise mimicking experimental conditions was also included. Note that the time-averaged cross-correlations are unaffected by the presence of 5%-20% bleed through under these conditions, whereas C(r, τ=0) is increased at short radii due to the presence of 10% and 20% bleed through.

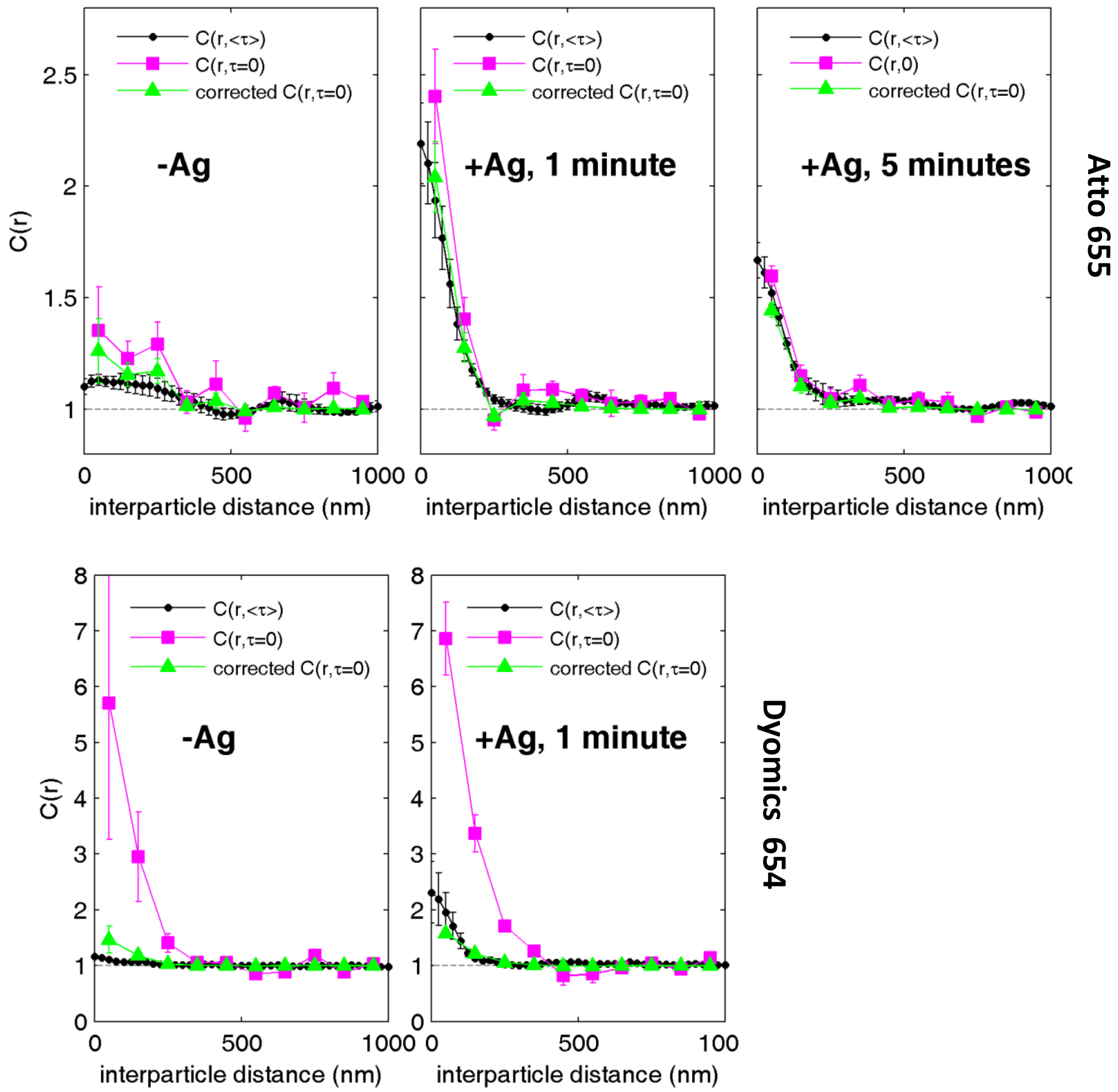

Supplementary Figure 9: Bleed-through leads to an increase in measured C(r,τ=0) that is roughly proportional to the magnitude of the actual correlation function. (top panels) Correlation functions averaged over chemically fixed cells (N=5 for left and center panel and N=4 for right panel) each chemically fixed either in the absence of antigen (left), after 1 minute of antigen stimulation (center), or after 5 minutes of antigen stimulation (right). Cells contain Atto 655 labeled BCR and mEos3.2 labeled Lyn. Bleed-through leads to a systematic increase in C(r, τ=0) compared to C(r, <τ>) that is roughly proportional to the magnitude of C(r, <τ>)-1. Here, the corrected C(r, τ=0) drawn is 0.75*(C(r, τ=0)-1)+1. **(bottom panels)** Correlation functions averaged over 3 cells chemically fixed either in the absence of antigen (left) or after 1 minute of antigen stimulation (right). Cells contain Dyomics 654 labeled BCR and mEos3.2 labeled Lyn. Here, the corrected C(r, τ=0) drawn is 0.1*(C(r, τ=0)-1)+1. Error bars represent σC.

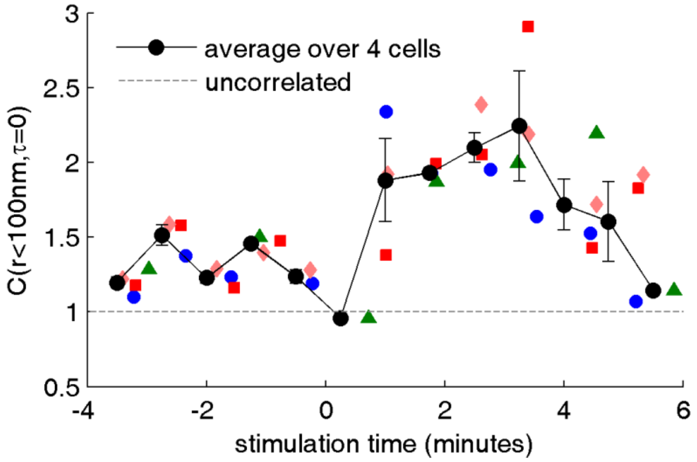

Supplementary Figure 10: C(r, τ=0) through time between BCR and Lyn is consistent in four independent cells. Time-course of the steady state cross-correlation of BCR and Lyn between 0 and 100 nm is plotted for 4 independent cells over steady-state time-intervals of 45 seconds. This time interval is approximately twice as large as that from the main text, due to differences in signal density between cells. Each cell is shown as a different color shape combination. The average of this time-course over all 4 cells is shown as solid black circles connected by lines, with error bars given by the standard error of the mean between the 4 cells. Zero stimulation time is when streptavidin was added to cluster and stimulate biotin-labeled B cell receptors.

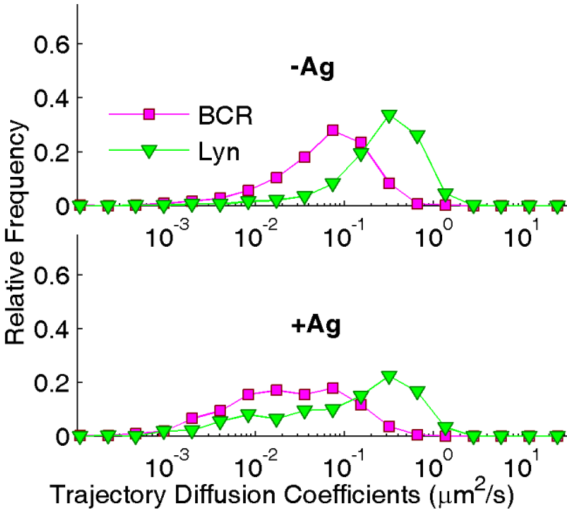

Supplementary Figure 11: Distributions of trajectory diffusion coefficients for both BCR and Lyn are shifted to lower values following antigen stimulation. Mean squared displacements (MSD) as a function of time-interval τ for individual trajectories of BCR and Lyn were fit to linear regressions for τ between 2 and 4 frames. The determined slope was divided by 4 to give the diffusion coefficient of that individual trajectory. The histogram of these diffusion coefficients is shown for BCR and Lyn in both unstimulated and stimulated frames, indicated by –Ag and +Ag respectively and corresponding to prior or after the addition of streptavidin to cluster and stimulate B cell receptors. Prior to stimulation, Lyn and BCR trajectory diffusion coefficients are centered at 0.4 $\mu m^2/s$ and 0.05 $\mu m^2/s$ respectively (upper panel). After stimulation, BCR trajectory diffusion constants shift to lower diffusion coefficients. This behavior is also seen in trajectory diffusion coefficients for Lyn, however the majority of diffusion coefficients from Lyn trajectories are unaffected by stimulation (lower panel).

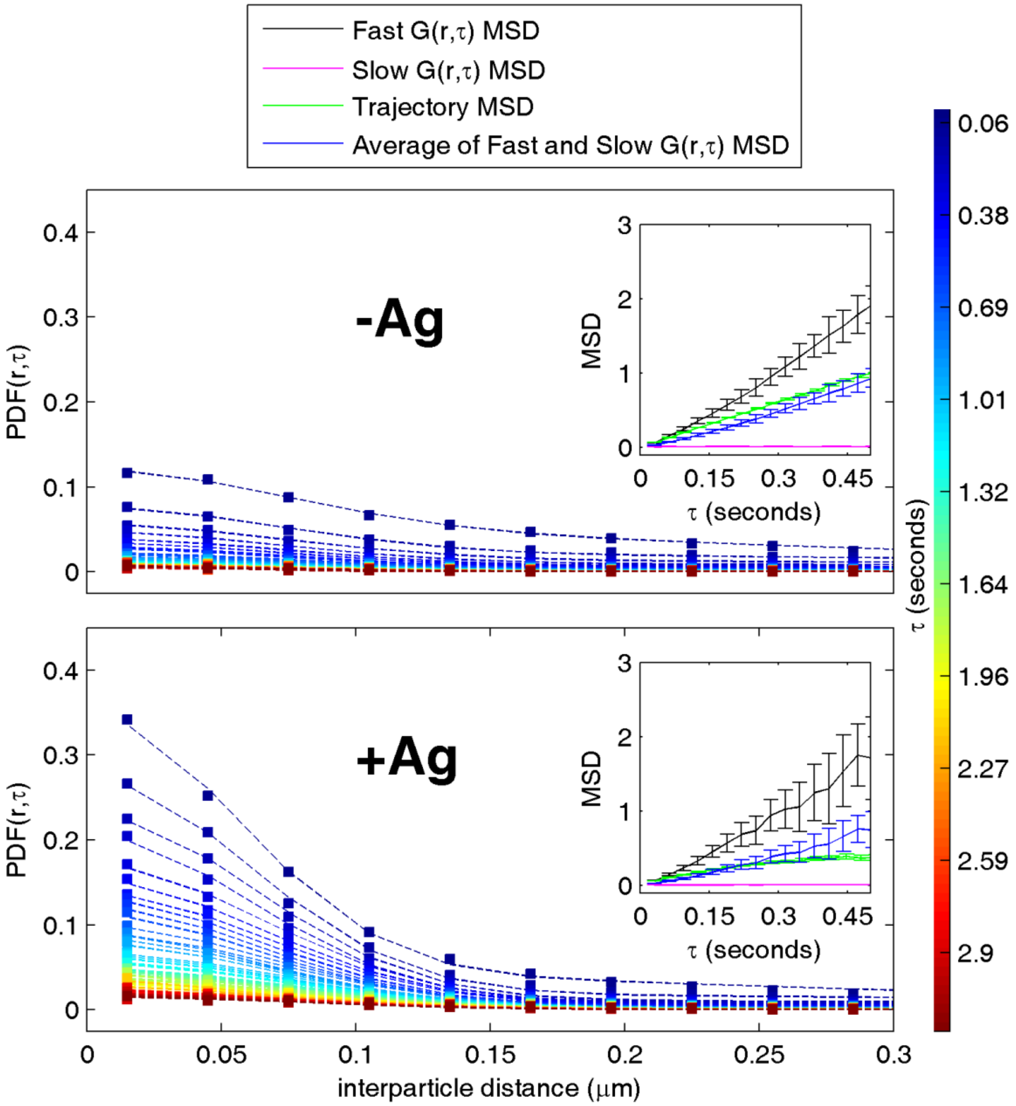

Supplementary Figure 12: MSDs determined from Gaussian fitting of PDF(r,τ) are in good agreement with MSDs determined from trajectory analysis. Experimentally determined probability distribution functions (PDFs) are determined from the auto-correlation of Lyn positions through time G(r,τ) as described in Methods. PDF(r,τ) is fit well by two Gaussian functions, where the Gaussian width reports on the MSD of that population of diffusers, and a parameter α reports the fraction of segments in the slow population. Raw PDF(r,τ) is shown as squares colored by τ, and Gaussian fits are shown as dotted lines also colored by τ. This MSD was used to quantify the diffusion of each population using MSD vs time curves, as shown in the inset. Black curves show the MSD from the fast population, magenta curves show the MSD from the slow population, and green curves show the MSD tabulated from trajectories. Blue curves show the average of the fast and slow population weighted by α. Colorbar on the right indicates the time lag between frames, τ, for the correlation functions G(r, τ). –Ag and +Ag, respectively, corresponding to prior or after the addition of streptavidin to cluster and stimulate B cell receptors. Error bars in black, magenta, and blue curves in inset are indicate one standard deviation of the MSD parameters from Gaussian fits to the PDF(r, τ). Error bars in the green curve are given by the standard error in the slope of the line fit to the 2nd through 4th points of the MSD tabulated from all trajectories in the time window.

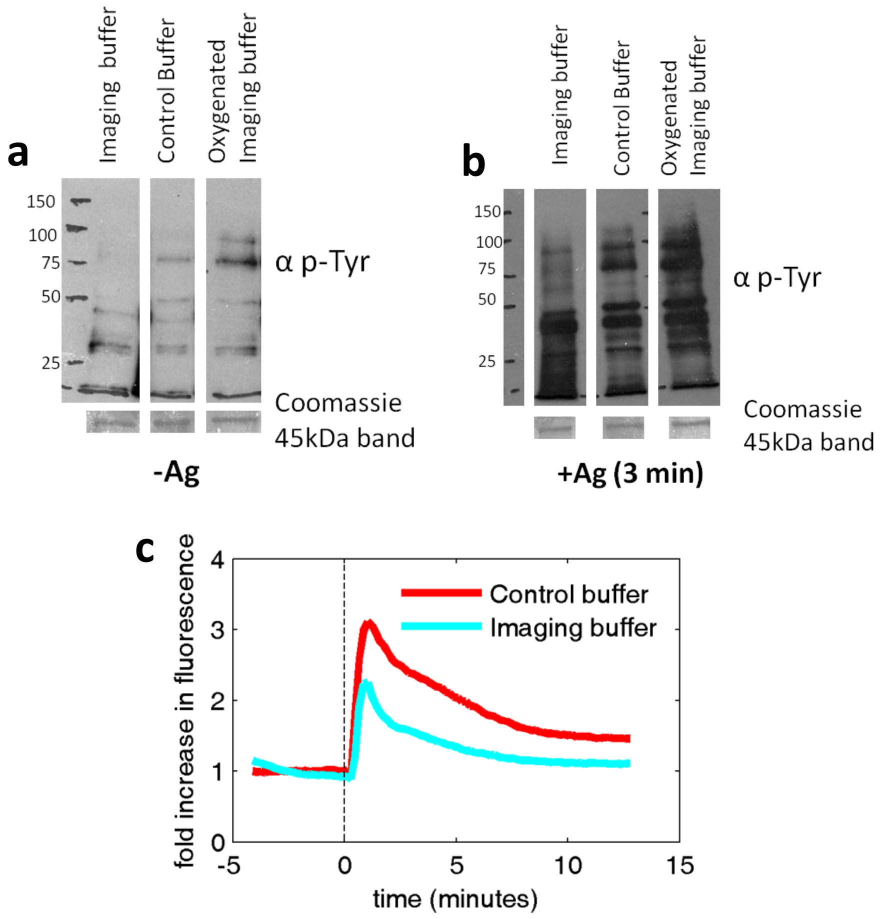

Supplementary Figure 13: Antigen induced phosphotyrosine and calcium mobilization is retained in CH27 cells under imaging conditions. a) Total phosphotyrosine western blot of CH27 lysates from unstimulated cells. IgM was labeled with IgM specific f(AB)$_1$ conjugated to biotin, and lysates were prepared from cells in either full imaging buffer, a control buffer, or the imaging buffer without oxygen scavenging enzymatic system as described in Materials and Methods. Molecular weight marker is shown at left. Coomassie stain of blot is shown at bottom, indicating total protein content for each lane is constant. Some higher molecular weight proteins have less phosphotyrosine staining in the full imaging buffer condition compared to the control buffer, however normal phosphotyrosine staining is recovered by using imaging buffer without the oxygen scavenging system. **b)** Phosphotyrosine western blot of CH27 lysates from cells 3 minutes after addition of 5 μg/ml streptavidin to stimulate BCR. Molecular weight marker is shown at left. Coomassie stain of blot is shown at bottom, indicating total protein content for each lane is constant. As in unstimulated cells, some higher molecular weight proteins have less phosphotyrosine staining in the imaging buffer condition compared to control buffer, however normal phosphotyrosine staining is recovered by using imaging buffer without the oxygen scavenging system. **c)** Cytoplasmic Ca^{2+} mobilization time-course from CH27 cells suspended in control and full imaging buffers, as indicated using Fluo-4 fluorescence intensity. Cells exhibit Ca^{2+} mobilization in both buffer conditions, although is attenuated in the full imaging buffer.
